# Effective non-drug interventions for improving outcomes and quality of maternal health care in sub-Saharan Africa: a systematic review

**DOI:** 10.1186/s13643-016-0305-6

**Published:** 2016-08-15

**Authors:** Frederick M. Wekesah, Chidozie E. Mbada, Adamson S. Muula, Caroline W. Kabiru, Stella K. Muthuri, Chimaraoke O. Izugbara

**Affiliations:** 1 African Population Health Research Center, 2nd Floor, APHRC Campus, Manga Close, Off Kirawa Road, Kitisuru, P. O. Box 10787, Nairobi, 00100 Kenya; 2 Julius Global Health, Julius Center for Health Sciences and Primary Care, Utrecht Medical Center, Utrecht Huispost Str. 6.131, P.O. Box 85500, 3508 GA Utrecht, Netherlands; 3 Department of Medical Rehabilitation, College of Health Sciences, Obafemi Awolowo University, Ile-Ife, Nigeria; 4 Department of Public Health, School of Public Health and Family Health, College of Medicine, University of Malawi, Private Bag 360, Chichiri, Blantyre, Malawi; 5 African Center for Public Health and Herbal Medicine, University of Malawi, Blantyre, Malawi

**Keywords:** Effectiveness, Interventions, Maternal health care, Quality, Morbidity, Mortality, Emergency obstetric care, Review, Sub-Saharan Africa

## Abstract

**Background:**

Many interventions have been implemented to improve maternal health outcomes in sub-Saharan Africa (SSA). Currently, however, systematic information on the effectiveness of these interventions remains scarce. We conducted a systematic review of published evidence on non-drug interventions that reported effectiveness in improving outcomes and quality of care in maternal health in SSA.

**Methods:**

African Journals Online, Bioline, MEDLINE, Ovid, Science Direct, and Scopus databases were searched for studies published in English between 2000 and 2015 and reporting on the effectiveness of interventions to improve quality and outcomes of maternal health care in SSA. Articles focusing on interventions that involved drug treatments, medications, or therapies were excluded. We present a narrative synthesis of the reported impact of these interventions on maternal morbidity and mortality outcomes as well as on other dimensions of the quality of maternal health care (as defined by the Institute of Medicine 2001 to comprise safety, effectiveness, efficiency, timeliness, patient centeredness, and equitability).

**Results:**

Seventy-three studies were included in this review. Non-drug interventions that directly or indirectly improved quality of maternal health and morbidity and mortality outcomes in SSA assumed a variety of forms including mobile and electronic health, financial incentives on the demand and supply side, facility-based clinical audits and maternal death reviews, health systems strengthening interventions, community mobilization and/or peer-based programs, home-based visits, counseling and health educational and promotional programs conducted by health care providers, transportation and/or communication and referrals for emergency obstetric care, prevention of mother-to-child transmission of HIV, and task shifting interventions. There was a preponderance of single facility and community-based studies whose effectiveness was difficult to assess.

**Conclusions:**

Many non-drug interventions have been implemented to improve maternal health care in SSA. These interventions have largely been health facility and/or community based. While the evidence on the effectiveness of interventions to improve maternal health is varied, study findings underscore the importance of implementing comprehensive interventions that strengthen different components of the health care systems, both in the community and at the health facilities, coupled with a supportive policy environment.

**Systematic review registration:**

PROSPERO CRD42015023750

**Electronic supplementary material:**

The online version of this article (doi:10.1186/s13643-016-0305-6) contains supplementary material, which is available to authorized users.

## Background

Sub-Saharan Africa (SSA) continues to experience very high rates of maternal morbidity and mortality [1–3]. The region has a lifetime risk of maternal deaths of 1 in 38 [3], with an estimated 530,000 maternal deaths occurring each year [4]. These deaths account for more than 90 % of all global maternal deaths [5]. The limited availability of basic and essential maternal health services, coupled with poor implementation of maternal health care policies and programs, continues to fuel the high maternal mortality ratio (MMR) in SSA [6].

The maternal health crisis facing the region has led to a proliferation of interventions to improve the quality of maternal health services and health outcomes [7–11]. These interventions take a variety of forms, including general health system strengthening through activities like training of health care providers on key skills such as emergency obstetric care (EmOC), prevention of mother-to-child transmission (PMTCT) of HIV, task shifting among health care workers, demand- and supply-side financial incentives, and mobile and electronic health interventions, among others. While evidence on the effectiveness of health interventions can support improvements in service delivery and promote population wellbeing [7, 10, 12, 13], there is a lack of critical and systematic analyses of the effectiveness of existing interventions that have been implemented in SSA to improve outcomes and quality of maternal health care.

In this review, we report on non-drug interventions and their effectiveness to improve outcomes and impact the quality of maternal health care in the region. Findings from this review will provide a basis for the design, delivery, and scale-up of programs aimed at improving the quality of care offered to women in region and consequently their health outcomes.

## Methods

We conducted a systematic literature search of papers on interventions aimed at improving the quality and effectiveness of maternal health care in SSA. The study protocol was registered in PROSPERO (CRD42015023750). We undertook a computer-aided search of online journal databases comprising African Journals Online (AJOL), Bioline, MEDLINE, Ovid, Science Direct, and Scopus. In the search, the following keywords and Boolean combinations were used: “maternity,” “maternal,” “maternal mortality,” “maternal deaths,” “morbidity,” and “antenatal” in combination with “Africa” or “sub-Saharan Africa” AND “quality” AND “effectiveness.” We further supplemented the searches with rigorous manual reviews of the reference lists of all included scientific publications for relevant additional literature. In this review, we defined a “research article” as an original piece of scientific work published in a peer-reviewed scientific journal. A “review article” was defined as a summary analysis of existing knowledge and insights into specific research areas published in a peer-reviewed scientific journal. To be included in the current review, articles had to be peer-reviewed papers published in English between 2000 and 2015. They also needed to report outcomes of non-drug interventions that sought to improve outcomes and quality of care in maternal health in SSA. Building on Glynn et al. [14], we defined non-drug interventions as those not related to or involving the use of drugs or medication, and directed to the individual (patient), members of her family, the health care providers, or the health care system with the aim of enhancing quality of care and improving maternal morbidity and mortality outcomes. Articles were excluded if they had no clearly stated design or evaluation methods. Two reviewers (FMW and CEM) independently identified articles for inclusion in the review. Disagreements on articles selected for inclusion were resolved through discussion and consensus among FMW, CEM, ASM and COI. Where consensus was still not possible, ASM had the casting decision.

### Criteria for study inclusion

Two hundred and fifteen articles on hospital- and community-based interventions aimed at improving outcomes and quality of care in maternal health in SSA were screened. One hundred and three of these articles were based on non-drug related interventions while 112 addressed drug or pharmacological-related interventions, including anti-retroviral therapy (ART) initiation in HIV pregnant women for prevention of mother-to-child transmission of HIV, use of misoprostol for labor induction during delivery, intermittent prophylaxis therapy—presumptive treatment of uncomplicated malaria during pregnancy (using drugs such as sulphadoxine-pyrimethamine and chloroquine), use of magnesium sulphate for management of eclampsia/preeclampsia, and provision of insecticide-treated nets to prevent malaria infection during pregnancy. Thirty out of the 103 articles on non-drug interventions were excluded for not reporting specific maternal outcomes, reporting process evaluation outcomes, or addressing cost effectiveness of such interventions. Fifteen of the excluded articles assessed nutritional supplementation among pregnant women, e.g., micronutrient supplementation and iron-fortified foods during pregnancy to prevent anemia and enhance safe delivery. In summary, a total of 142 studies were excluded from the current review.

Seventy-three non-drug intervention studies were included in this review. These included 24 randomized controlled trials; 16 retrospective exploratory, cohort, or comparative studies; 12 prospective exploratory, cohort, or comparative studies; 10 quasi-experimental studies, 5 studies that analyzed cross-sectional data from cluster randomized trials; one time series observational study with a control arm; one case cohort study; three cross-sectional analyses of pre- and post-intervention or case control studies; and one non-randomized pre-post study.

**Fig. 1:**
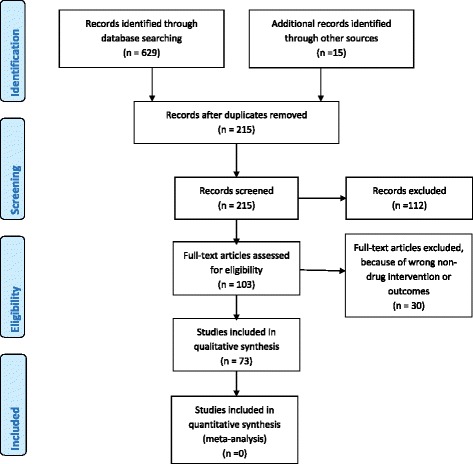
PRISMA 2009 Flow Diagram for included studies

The PRISMA flow diagram, 2009 [15], showing the process of selecting studies for inclusion is in this review is included (Fig. 1) together with a PRISMA checklist [15] for items considered while conducting this review (Additional file 1).

The review employed the modified Newcastle-Ottawa scale for case/cohort/cross-sectional studies [16] and the 5-point Jadad scale for experimental studies/RCTs [17] for quality assessment. The information on the level of the quality of evidence from the included studies is shown in the Table 1, ranging from level I to V (low to high quality). The quality checks focused on (a) representativeness of the population and population characteristics, (b) information about the study design, (c) ascertainment of exposure and intervention, and (d) use of standard definitions for main outcome measures and denominators used (e.g., for deaths). From each paper, we extracted information on issues that potentially affected the observed outcomes (i.e., frequency and duration of data collection), proportion of refusals and loss to follow-up, and sample sizes. In addition, we extracted general study information (e.g., year the study was published and authors) and the primary health outcomes for the intervention. From the findings of studies included in the review, we checked for conclusions regarding the effectiveness of the intervention in improving one or more aspects of the quality of maternal health care as defined by the Institute of Medicine 2001 (“Crossing the quality chasm: A new health system for the 21st century”) as having the following key dimensions: safety, efficiency, effectiveness, equitability, patient centeredness, and timeliness. A narrative synthesis highlights findings from the included studies. Due to large heterogeneity in study methodologies, outcomes, outputs, and processes, a quantitative/meta-analysis analysis was not possible.

**Table 1:** Findings of the effective non-drug interventions for improving outcomes and quality of care in maternal health in sub-Saharan Africa (*n* = 73)

Intervention	Studies analyzed	IOM aspect of quality of care addressed	Method of implementation	Impact and effect of the intervention on beneficiaries	Remark	Limitations and strengths
1. Mobile and electronic health interventions.	1. Ngabo et al. 2012 [24]; (level III); 2. Lund et al. 2014 [21]; (level II); 3. Oyeyemi et al. 2014 [23] (level IV); 4. Horner et al. 2013 [26]; (level III); 5. Dalaba et al. 2015 [25]; (level III);	Patient centeredness; equitability; timeliness; efficiency; safety	1. Provision of mobile phones to pregnant women; 2. Implementing an SMS-alert program; 3. Provision of mobile phone to CHWs and service vouchers to pregnant mothers; 4. Use of electronic decision support system to improve compliance of health workers to the existing maternity care protocols; 5. Interactive communication systems between a community health worker (CHW) following mother-infant pairs in their community, a national centralized database, and the health facility and in case of an emergency alert, an ambulance operator.	Provides two and three-way communication for action between women and the health system; Increased uptake of primary health care services; Increased number of women receiving preventive health services; Increase in antenatal care attendance; Increased number of women attending ANC late into pregnancy; Improved ambulance requests and referral system; Increased facility-based deliveries; Overall improvement in compliance to maternity care guidelines.	Mobile phone interventions may contribute towards increased access to maternal health services and facility utilization which is essential for improved maternal and newborn health. Use of electronic health interventions is effective in improving health workers’ compliance to maternity care protocols.	Lack or interrupted electricity and mobile network failure were the major challenges to mHealth use. The paper by Oyeyemi et al. (2014) [23] was based on a case control study thus limited in strength of its evidence; The interventions were often small in scale and at high risk of possible dilution effect between intervention facilities and controls which were not controlled for in these studies.
2. Financial incentives including user fee exemptions, payment for performance, vouchers and community-based insurance schemes.	1. Bellows et al. 2012 [32]; (level III); 2. Fournier et al. 2014 [34]; (level II); 3. Richard et al. 2008 [58]; (level II); 4. Watts et al. 2015 [37] ; (level II); 5. Amendah et al. 2013 [33]; (level V); 6. Obare et al. 2013 [36]; (level V); 7. Obare et al. 2014 [35]; (level V); 8. Adinma et al. 2011 [40]; (level V); 9. Ezugwu et al. 2011 [41]; (level III); 10. Frimpong et al. 2014 [38]; (level III); 11. Smith et al. 2008 [43]; (level II); 12. Basinga et al. 2011 [44]; (level II); 13. Bonfrer et al. 2014 [42]; (level V); 14. Alfonso et al. 2015 [39]; (level II);	Equitability; effectiveness; safety; efficiency; timeliness; patient centeredness	1. Output-based approach voucher (covering ANC visits, facility-based delivery including caesarean section (CS) and postnatal care for mother and child); 2. Fee exemption (including for caesarean sections); 3. Cost-sharing programs between the community and the health care system/facilities; 4. Community-based insurance schemes; 5. Exemptions from premiums payable to CBHI; 6. Payment for performance (P4P); 7. Performance-based financing (PBF).	Incentivizes patients and health care workers; Improves quality of services provided; Increase access to spectrum of services available at the health facilities; Enhances facility-based deliveries; Increase in caesarean deliveries (mostly in cities than rural settings); Reduction in maternal morbidity and mortality; Voucher program improved poor women access to facility-based delivery; Births occurring at home declined; Premium exemptions led to increased registration with CBHI; ANC attendance; institutional deliveries; PBF improved the utilization and quality of maternal and child care in Burundi; PBF improves quality of maternal care without additional costs to the patients.	Financial incentives can enhance demand for facility-based maternal deliveries and also provide a platform for supply of quality maternal health services.	Evidence on the effects of financial incentives on maternal outcomes and equity is weak. Some of the studies were based on population-based cohort data, others have small sample sizes and were without a comparison or control.
3. Clinical audits, maternal death reviews and feedback	1. Browne et al. 2015 [126]; (level V); 2. Hunyinbo et al. 2008 [49]; (level II); 3. Pirkle et al. 2013 [111]; (level II); 4. Zongo et al. 2015 [50]; (level II); 5. Kongnyuy et al. 2008 [57]; (level III); 6. Kongnyuy et al. 2009a [55]; (level III); 7. Kongnyuy et al. 2009b [56]; (level III); 8. Igwegbe et al. 2012 [127]; (level III); 9. Ediau et al. 2013 [54]; (level II); 10. Strand et al. 2009 [128]; (level III); 11. Dumont et al. 2005 [53]; (level III); 12. Dumont et al. 2006 [59]; (level III); 13. van der Akker et al. 2009 [52]; (level III); 14. van der Akker et al. 2011 [51]; (level III).	Safety; efficiency; effectiveness; timeliness; patient centeredness	1. Criterion-based clinical audits (CBCA); 2. Maternal death reviews (MDR) and feedback.	Greater clinical examination and postpartum monitoring practices; Increased diagnosis of maternal morbidity; Decrease in aggregate case fatality rate from hemorrhage, eclampsia, obstructed labor and genital tract sepsis; Increased health facility deliveries and caesarean delivery; Improved quality of care; Reduction in maternal morbidity and mortality; Increased provision and quality of EmOC; Increased facility deliveries; CBCA helped improve documentation especially cases notes and maternity registers.	Clinical audit approaches in obstetric care are effective in improving quality of clinical care in resource-poor settings and consequently reduce maternal morbidity and mortality.	CBCA approaches and reports are limited to health facilities and do not provide a comprehensive overview of all maternal deaths in the community; There is also some variability in the standard checklists used in clinical audits among studies.
4. Comprehensive interventions targeting health systems strengthening—training of health care workers, infrastructural upgrading and provision of equipment and medical supplies.	1. Ameh et al. 2012 [67]; (level II); 2. Dumont et al. 2013 [68]; (level II); 3. Spitzer et al. 2014 [69]; (level III); 4. Sorensen et al. 2010 [70]; (level III); 5. Richard et al. 2008 [58]; (level II); 6. Sibley et al. 2014 [74]; (level V); 7. Kayongo et al. 2006 [65]; (level II); 8. Kayongo et al. 2006 [66]; (level II); 9. Brazier et al. 2009 [79]; (level II); 10. Santos et al. 2006 [81]; (level II); 11. Findley et al. 2013 [71]; (level II); 12. Mekbib et al. 2003 [72]; (level II); 13. Srofenyoh et al. 2012 [84]; (level III); 14. Agha, 2010 [83]; (level II); 15. Hounton et al. 2008 [76]; (level II); 16. Worku et al. 2014 [75]; (level III); 17. Richard et al. 2008 [58]; (level II); 18. Ediau et al. 2013 [54]; (level II); 19. Warren et al. 2010 [77]; (level II); 20. Galadanci et al. 2011 [78]; (level III); 21. Geerts et al. 2004 [82]; (level III); 22. Doherty et al. 2009 [80]; (level III).	Safety; efficiency; effectiveness; equitability	1. Training of health care workers in basic and comprehensive EmOC; 2. Training in skills for MDR; 3. Training in Advanced Life Support in Obstetrics care (ALSO); 4. Health facility and infrastructure renovations; 5. Strengthening existing referral systems; 6. Supportive supervision; 7. Logistics for supplies, equipment and drugs, record keeping, monitoring and evaluation, MDR.	Availability and improvement in quality of EmOC; Improved knowledge and confidence in carrying out clinical audits; Decreased postpartum hemorrhage rates; Reduction in hospital-based maternal morbidity and mortality; Significant decrease in neonatal deaths before mother and child discharge; Improvement in access to quality caesarean deliveries; Increased use of facility-based maternity care and institutional deliveries; Increase in fully functional EmOC facilities; Steady increase in number of complications treated; Decline in deaths from obstetric complications; Reduction in the aggregate case fatality rate (CFR).	Health systems capacity strengthening at the facility and community level led to building sustainable human resources and increased coverage for maternal health services, hence improved quality of care and reduction in maternal mortality.	Use of competent non-medically qualified persons (NMQP) incur lower remunerations and training costs compared with physicians; High level of supervision is required for NMQP to offer specialized services; Training in ALSO had no effect on the management of prolonged labor.
5. Community mobilization and peer-based Interventions	1. Colbourn et al. 2013 [91]; (level II); 2. Mushi et al. 2010 [92]; (level II); 3. Richter et al. 2014 [90]; (level II); 4. Wangalwa et al. 2012 [93]; (level III); 5. Tesfaye et al. 2014 [73]; (level III); 6. Ensor et al. 2014 [95]; (level II); 7. Hounton et al. 2009 [96]; Level II); 8. Ediau et al. 2013 [54] (level II).	Effectiveness; timeliness; patient centeredness; equitability; efficiency; safety	1. Community mobilization through women’s groups; 2. Skilled birth attendants; 3. Training health extension workers; 4. Training and deployment of community health development agents; 5. Traditional birth attendants (TBAs); 6. Family and community members meetings on health care; 7. Trained volunteers to provide health care at the community; 8. Village health teams; 9. Peer mentors who women are living with HIV (WLH) to support pregnant WLH.	Community Mobilization: Promotes utilization of obstetric care; Increase in first and subsequent ANC attendance and postnatal care; Increase in health facility use and deliveries; Increase in level of health information about danger signs and risk factors in pregnancy; Reduction in perinatal mortality rates; The number of male partners counseled, tested and given results together with their wives at first ANC visit rose. Peer mentorship: Higher likelihood to complete both maternal and infant ARV; Increase adherence to all PMTCT tasks; Greater likelihood to ask partners to test for HIV; Less likelihood to report depressed mood; Deliveries with skilled attendant significantly increased; Significant increase in attendance of at least four ANC visits, deliveries by skilled birth attendants; Number of pregnant women attending first ANC visit significantly increased; The number of pregnant women counseled, tested and given results for HIV during the first ANC attendance significantly rose.	Community mobilization interventions to reduce maternal mortality improved equitable access maternal health services and thus and reduce maternal mortality. Also, peer mentors interventions by WLH for pregnant WLH led significant overall benefits compared to standard care.	Community mobilization interventions cannot substitute for a formal health system but serves as a veritable platform to bridge inequities in maternal health care access and utilization.
6. Home visits and counseling by health care workers.	1. le Roux et al. 2013 [100]; (level II); 2. Lewycka et al. 2013 [101]; (level II); 3. Magoma et al. 2013 [94]; (level II); 4. Rotheram-Borus et al. 2014 [89]; (level II); 5. Jennings et al. 2010 [102]; (level II);	Patient centeredness; Equitability	1. Home visit by community health workers (CHWs); 2. Home visit by peer groups; 3. Use of women’s groups and volunteer peer counselor for health education; 4. Birth plans counseling and health education; 5. Job aids counseling by nurses and midwives; 6. counseling by lay nurse aides.	Improved adherence to condom use among pregnant WLH; Increased uptake of skilled delivery and post-delivery care; Improvement in birth preparedness among women; Danger sign recognition by pregnant women; Skilled deliveries and newborn care; Enhanced maternal and child outcomes; Mothers in the intervention group were more likely to use condoms consistently.	Home visits and counseling by community health workers can help reduce maternal mortality in resource-limited settings with limited access to facility-based maternal health care.	Home visit interventions most often require antenatal visits to initiate contacts with the pregnant women; High intervention coverage may be required in order to achieve significant reductions in maternal mortality; Methods on conducts of home visit intervention may vary widely across countries and regions and as such may affect the external validity of the findings in this review.
7. Emergency transportation, communication and referrals for obstetric care.	1. Mucunguzi et al. 2014 [103]; (level II); 2. Schoon, 2013 [104]; (level II); 3. Tayler-Smith et al. 2013 [105]; (level III); 4. Fournier et al. 2009 [106]; (level III);	Equitability; timeliness; safety	1. Transportation and communication intervention; 2. Inter-facility transportation program; 3. Patient transfer system to emergency obstetric care facilities; 4. Maternity referral system for emergency obstetric health services; 5. Voucher scheme plus round trip transportation.	Increase in hospital deliveries; Reduction in the average hospital stillbirths; Increased access to emergency obstetric care and caesarean sections; Increased institutional deliveries; Reduced risk of death form an obstetric emergency; Decrease in maternal mortality rates; Reliable communication and transport services increased access to and utilization of maternal health services, particularly in caesarean section deliveries; Effective and prompt inter-facility transport of patients with pregnancy complications to an appropriate facility resulted in a reduction of maternal mortality.	Emergency transport can play an important role in reducing maternal mortality and morbidity; Effective communication systems, transport services and prompt referrals to appropriate facilities will increase access to and utilization of maternal health services, particularly caesarean delivery services and result in a reduction of maternal mortality.	Emergency transportation of pregnant women to maternal health care facilities and subsequent referral often have reliance on mobile phones; Referral to emergency obstetric care in most rural settings is beset with challenges such as difficult topographical landscape, limited number of vehicles, and the spread of maternal health care facilities.
8. Prevention of mother-to-child transmission (PMTCT) of HIV	1. Byamugisha et al. 2011 [109]; (level II); 2. Delvaux et al. 2008 [112]; (level II); 3. Pirkle et al. 2014 [110]; (level V); 4. Ediau et al. 2013 [54]; (level II).	Safety; effectiveness	1. Training of health care workers on strategies to enhance the PMTCT; 2. Equipping of health facilities with medical supplies used in PMTCT; 3. Community mobilization to encourage male partners’ acceptance of HIV testing through joint attendance to clinics with women.	Increase in the number of pregnant women attending first ANC visit; Number of male partners counseled, tested and given results for HIV together with their wives at first ANC visit also rose significantly; Significant rise in the number of pregnant women delivering in the health facilities.	PMTCT services involving capacity training of health care workers on PMTCT provision, availability of medical supplies used in PMTCT and community mobilization to encourage male partners’ acceptance of HIV testing improves maternal health indicators in SSA.	This review considered only the non-drug components of PMTCT. Therefore, it is difficult to isolate the specific effects of the non-drug components, from the cascade of PMTCT services.
9. Task shifting interventions	1. Gessessew et al. 2011 [123]; (level III); 2. Jennings et al. 2011 [122]; (level III).	Efficiency; effectiveness; equitability	1. Antenatal counseling by lay nurse aids; 2. Performance of emergency caesarean sections by non-physician clinicians (NPCs).	Improved maternal knowledge on prenatal care, birth preparedness and recognition of danger signs among women being counseled; Reduced hospital-based maternal and fetal deaths arising from obstetric complications; Postoperative outcomes achieved under the care of non-medically qualified persons were similar to those attained by physicians.	Task shifting interventions may improve cost effective access to and availability of maternal health care services without compromising the essential maternal health service delivery or patient outcomes.	Task shifting interventions often employ competent non-medically qualified persons who incur lower remunerations and training costs compared with physicians, however, high level of supervision is required for them to deliver the expected impact services.

## Results and discussion

The range of interventions to improve quality of maternal care and maternal outcomes included in this paper is summarized in Table 1. The interventions were broadly classified into mobile and electronic health interventions (*n* = 5; 7 %); financial incentives in the form of user fee exemptions and payments to health facilities based on performance (*n* = 13; 18 %); clinical audits (*n* = 13; 18 %); health systems and infrastructure development (*n* = 23; 32 %); community mobilization and peer-based programs (*n* = 8; 11 %); home visits and counseling and educational and health promotion programs (*n* = 5; 7 %); emergency transportation, communication, and referrals (*n* = 4; 6 %); PMTCT (*n* = 4; 6 %); and task shifting interventions (*n* = 2; 3 %). Some of the studies covered in the review were included in more than one category. The summary of results of the review is presented in Table 1. Below, we highlight the key findings of the studies within each of the broad classifications.

1. Mobile and electronic health interventions

Mobile health (mHealth) refers to the application of wireless, portable information and communication technologies to support health and health care [18, 19]. These interventions often involve the use of devices such as phones, computers, personal digital assistants, and digital point-of-care testing devices [20]. Although evidence of the potential value of mHealth for maternal health is just emerging, donors and development agencies continue to give considerable attention to it as a means of improving maternal health [21, 22].

Three studies on mHealth were included in this review. These studies were conducted in Nigeria [23], Rwanda [24], and Zanzibar [21]. The mHealth interventions sought to provide two- or three-way communication between women and the health care system, through provision of mobile phone messages reminding pregnant women to attend clinic days. Overall, evaluations of the mHealth interventions show that they improved utilization of, and access to maternal health services, as well as women’s attendance at primary health care facilities and services such as antenatal care (ANC). In Zanzibar, the provision of mobile phones, together with redeemable vouchers for pregnant women, reportedly improved the timing of ANC services as well as the number of women seeking and receiving preventive health services, attending ANC late in pregnancy (i.e., more ANC visits and those treated for antepartum complications). In Rwanda, the short message service (SMS)-based alert program called the Rapid SMS-MCH was used to monitor pregnancy by allowing a three-way interactive communication among a community health worker (CHW) following pregnant women in the community, a health care worker at a health facility, and an ambulance driver who would be called in to facilitate emergency transport for obstetric care. The project led to an increase in facility-based deliveries from 72 to 92 % at the end of a 12-month pilot phase.

An electronic clinical health decision system (eCDSS) implemented in Ghana [25] and the Basic Antenatal Care Information System (Bacis) implemented in South Africa [26] were shown to improve health workers’ compliance with established maternity care protocols. In Ghana, health workers’ compliance with maternal care protocols reportedly increased the detection of pregnancy complications during ANC in the health centers, consequently decreasing the number of labor-related complications. The *Bacis* system in South Africa improved health workers’ responsiveness and quality of services offered to their patients. It also improved the promptness and quality of services offered to mothers younger than 18 years and their retention in ANC beyond week 20. On final assessment, the *Bacis* program showed minimal health data input error (13.2 %) compared to the District Health Information System (DHIS) that was in use (25 %).

Mobile health and electronic health interventions may improve health care processes through lower failed appointments for services like ANC and quicker diagnosis and treatment of obstetric complications. Unreliable electricity infrastructure and supply and mobile network failure were the major challenges to the mHealth and e-health initiatives identified in this review. The interventions considered were also small in scale and at high risk of dilution of possible impact between intervention and control communities.

2. Financial incentives

Financial incentives to improve access and utilization of maternal health care and service delivery have also been implemented in a number of countries in SSA [27–29]. Interventions involving financial incentives take the form of supply-side and demand-side incentives and seek to enhance equitable access to health care while improving the market supply and quality of services available to underserved populations [30, 31]. Supply-side incentives include pay for performance (P4P), performance-based financing (PBF), and various community-based insurance schemes (CBHI) and while demand-side incentives include vouchers that can be redeemed for health services, user fee exemptions, conditional cash transfers, and subsidies or cost-sharing arrangements.

We reviewed 12 studies that reported on the effectiveness of demand-side financial incentives including user fee exemptions (majorly for caesarean section deliveries), cost-sharing programs between the public and the health care facilities, and output-based approach (OBA) vouchers covering costs for ANC visits, and facility-based deliveries. Other demand-side financial incentives implemented were vouchers redeemable for emergency transport to facilitate timely movement of mothers requiring emergency obstetric care and vouchers to health service providers to redeem costs incurred for providing delivery services in their facilities. The OBA vouchers supported targeted subsidies to underserved populations in the urban slums of Nairobi increased facility-based deliveries [32, 33]. Results showed that beneficiaries of the OBA voucher were more likely to deliver a subsequent child in a health facility compared to non-beneficiaries. Fee exemption for caesarean sections increased caesarean birth rates in cities in Mali, which reportedly reduced the risk of maternal deaths from obstructed labor [34]. The Safe Motherhood Initiative (SMI) provided vouchers to facilitate access to health facility deliveries for women in rural Kenya. This program significantly decreased the number of home births by about 10 % and added to the number of facility-based deliveries by a similar margin [35–37]. In Ghana, exemptions from community-based health insurance program premiums increased the registration of female community members, enhancing uptake of health services and leading to improved ANC attendance, and facility-based deliveries [38]. The premium exemptions also increased access to a spectrum of other services offered at health facilities, including clinical and diagnostic services during ANC visits, counseling on safe motherhood, education on recognition of pregnancy-related danger signs, and vaccination against tetanus. A voucher system that catered for costs for ANC services and hospital delivery in Uganda raised demand for facility-based deliveries by 52.3 %, of which about 9 % were new health facility users [39]. In Nigeria, a co-financing program for maternal health between the government and the community resulted in a 60 percentage point increase in the utilization of maternal health services (from 26.7 to 85.6 %) [40], while a user fee exemption program in the same country resulted in increased uptake of institutional deliveries by up to 88 %, resulting in a decline in institutional MMR from 532 to 371 per 100,000 live births [41].

Burundi [41, 42], Ghana, Mali and Senegal [38, 43], Rwanda [44], and Uganda [39] have implemented community-based health insurance schemes, in some cases, combined with the exemption of pregnant women from the premiums payable to these schemes. The P4P scheme in Rwanda resulted in a 23 % increase in the number of institutional deliveries while in Burundi, the PBF initiative led to significant improvements in the quality of ANC services received by women at health facilities that participated in the intervention.

Financial incentives have the potential to increase access to and utilization of maternal health services especially among the poor, thereby facilitating facility-based deliveries, use of skilled care, and reduction of maternal mortality [45]. However, evidence on the impact of financial incentives on the quality of and equitable access to maternal health care in SSA is inconclusive. Some of the studies reviewed relied on small sample sizes or did not have comparison group and therefore did not offer high-quality evidence.

3. Clinical audits

Several studies examined the use of audit tools including criterion-based clinical audits (CBCA) and maternal death reviews (MDR) to improve the quality of maternal care in health facilities. CBCA is a tool for measuring and improving the quality of obstetric care at health facilities with a focus on five life-threatening obstetric complications: hemorrhage, eclampsia, genital tract infection, obstructed labor, and uterine rupture [46, 47]. MDR, on the other hand, employs a qualitative approach to investigate the causes of and circumstances surrounding maternal deaths at health facilities [48]. Both approaches are combined with feedback and target setting sessions with health care providers, to improve the management of obstetric cases, improve quality of care, and reduce maternal mortality. The main element of CBCA is that performance is audited to ascertain whether guidelines were followed. The tool facilitates improvements on areas of weakness in situations where the right processes in the management of obstetric complications are not followed.

CBCA implemented in a health facility in Nigeria [49] resulted in an improvement of the overall care for obstetric complications by 20 % (from 61 to 81 %) for obstetric hemorrhage; 54 to 90 % for eclampsia; 82 to 94 % for obstructed labor and from 66 to 85 % for genital tract sepsis. Zongo et al. [50] reported that women treated at facilities where CBCA was implemented in Mali and Senegal had better clinical examination and postpartum monitoring practices. Similarly, in a study in Malawi [51, 52], the use of CBCA decreased the incidence of uterine rupture by 68 % (from 19.2 to 6.1/1000 deliveries), improved the diagnosis of maternal morbidity, and increased the number of women receiving blood transfusions and caesarean deliveries. Other studies reported that CBCA decreased the aggregate obstetric case fatality rate (CFR) by 33 % in Senegal [53] and contributed to an increase in first ANC visits, facility deliveries (from 31.8 to 34.7 %), and the quality of women-friendly services and satisfaction of women (by 9 % in Uganda) [54]. In addition, CBCA was shown to reduce institutional MMR from 250 to 182 per 100,000 live births, decrease the CFR from 3.7 to 1.5 % [55–57], improve documentation by health care workers of case notes and maternity registers, and increase caesarean delivery rate from 1.9 to 3.3 % in Burkina Faso [58].

The multifaceted QUARITE (quality of care, risk management, and technology in obstetrics) study conducted in Mali and Senegal [50] and another study on facility-based maternal death reviews conducted in Senegal [59] reported that MDR and on-site training of health care providers on EmOC reduced maternal mortality among high-risk women who delivered through caesarean section. In addition, MDR reportedly decreased obstetric complications and the overall hospital-based MMR by a massive 410 in 100,000 and led to a drop in the proportion of women who had not received any ANC services from around 11 to 4 % at the end of the intervention [59].

Although CBCA and MDR have been shown to be effective in improving the quality of EmOC and the management of obstetric cases, leading to a reduction in obstetric case fatality rates, their strength of evidence is limited. The techniques are often health facility-based interventions and do not provide a comprehensive overview of all cases of maternal deaths in a community. In addition, variability in standard checklists used in clinical audits potentially limits the replicability and comparability of clinical audits.

4. Comprehensive interventions targeting health systems strengthening—training of health care workers, infrastructural upgrading, and provision of equipment and medical supplies

Strengthening health systems was considered a key pillar to achieving the Millennium Development Goals (MDGs) and will still be key in achieving the Sustainable Development Goals (SDGs) [60]. Inequitable access to quality sexual and reproductive health care in SSA is exacerbated by acute shortage and unequal distribution of skilled and specialized health personnel such as physicians, midwives, and nurses [61, 62]. Efforts to address shortages in specialized care, and inequities in the distribution of skilled health personnel, have sought to build the capacity of available maternal health services providers in the provision of basic and comprehensive emergency obstetric care and in conducting vital quality assurance exercises, such as maternal death reviews [63, 64].

The CARE/AMDD (Averting Maternal Death and Disability) program collaboration is a comprehensive intervention implemented in three high maternal mortality countries in Africa (Rwanda, Tanzania, and Ethiopia) aimed at increasing the availability and quality of EmOC services [65, 66]. The program supported the three countries mainly by training health care workers to provide EmOC services, strengthening existing health information systems (HIS) to monitor change, and identifying gaps in quality of care. Technical leaders and policy makers in these countries were involved in the development and formative stages of internal quality review systems, to establish nationally acceptable standards and guidelines for maternal health care. Following implementation, there was a reported increase in the availability and utilization of EmOC in Tanzania, where the met need for EmoC—defined as a percent of all women with major direct obstetric complications who are treated in a health facility providing emergency obstetric care in a given reference period—was shown to have increased slightly from 14 to 19 % over 4 years, while in Rwanda it reportedly increased met need for EmOC from 16 to 25 %. Obstetric case fatality rates were also shown to have declined by between 30 and 50 % in all the three countries. There was a reported general increase in the level of preparedness for emergencies and the ability of health care workers in these countries to manage common obstetric complications according to evidence-based practices.

Training of health care workers on emergency obstetric care was shown to have impacted positively on the availability and quality of EmOC and improved delivery and care skills among midwives in Somalia [67]. It was also associated with improved knowledge and confidence in carrying out clinical audits among health care professionals. The program reportedly reduced hospital-based mortality in first-level referral hospitals in the country [68]. In Kenya, training of health care workers on EmOC was associated with decreased rates of postpartum hemorrhage [69]. Similarly, health providers trained on providing care for postpartum hemorrhage (PPH) under the Advanced Life Support in Obstetrics (ALSO) program in Tanzania led to a reduction in the incidence of PPH from 32.9 to 18.2 % and that of severe PPH from 9.2 to 4.3 % [70].

The Integrated Maternal Newborn and Child Health Program in Nigeria (an intervention that was based on a network of CHWs, who connect between households and the health facilities) [71]; the FIGO Save the Mothers Initiative in Ethiopia that aimed to establish basic and comprehensive EmOC in order to increase the availability and utilization of quality obstetric care based on UN indicators; and the Maternal Health in Ethiopia Partnership (MaNHEP) in Ethiopia that was based on community-oriented model for improving maternal and newborn health [72–74] were all reported to have improved access and the delivery of quality caesarean section to women and decreased obstetric case fatality rates. A skilled care initiative was also shown to have led to a 34 % decline in pregnancy-related mortality and the reduction in adverse pregnancy outcomes (obstetric complications and deaths) in Ethiopia and in Burkina Faso, respectively [75, 76].

The quality improvement packages, incorporating, among other things, quality assurance and provision of equipment were shown to have improved performance of health care providers in counseling women on recognizing and managing maternal complications in Kenya [77]. In Nigeria, an obstetric service quality assurance [78], together with improvements in infrastructure, provision of equipment, and continuous maternal and fetal data collection, analysis, and feedback, was shown to have lowered mean institutional MMR from 1790 per 100,000 births in 2008 to 940 per 100,000 births in 2009.

In Burkina Faso, health care providers were trained on routines and the provision of EmOC services, available referral systems were strengthened, health infrastructure was upgraded, and equipment as well as medical supplies including essential drugs were provided. This strengthening of community and health facility systems increased facility-based deliveries from 24 to 56 % and attendance by skilled care providers from 33 to 65 % among the women who reported an obstetric complication. The authors reported an increase in first ANC visits by women and their partners [58, 79]. A study on a participatory quality improvement intervention conducted in 21 health facilities in South Africa enhanced the uptake of CD4 testing from 40 to 97 %. In addition, it showed an improved uptake of maternal and infant nevirapine from 57 to 96 % and 15 to 68 % respectively, as well as an increase in the 6-week polymerase chain reaction (PCR) testing from 24 to 68 % [80], adding to efforts to prevent mother-to-child transmission of HIV.

Improvements in infrastructure, human resource development, transportation and communication systems, and management of health facilities were associated with the growth of fully functional EmOC facilities from 4 to 18 in Mozambique, a steady increase in number of complications treated, and a 50 % decline in deaths from obstetric complications. The met need for EmOC also reportedly increased 3-fold (from 11.3 to 32.8 %) in all facilities while births from caesarean section deliveries increased steadily, doubling at the end of the intervention. There was also a reduction of aggregate obstetric CFR by almost half (2.9 to 1.6 %); with concomitant decline in the deaths from obstetric hemorrhage, obstructed labor, and postpartum sepsis [81]. A community-based basic ultrasound service significantly reduced referrals to a regional center for fetal surveillance and delivery in South Africa [82]. In Uganda, health facility capacity strengthening was shown to have significantly improved the number of pregnant women delivering in the health facility from 55.2 to 99.3 % following the provision of *mama-kits* (delivery kits) [54]. A quality improvement package incorporating quality assurance for health facilities was linked to enhanced counseling and technical aspects of service provision in family planning and antenatal care in Uganda [83], and in Ghana [84], a 34 % decrease in maternal mortality, a decrease from 3.1 to 1.1 % of case fatality rates for preeclampsia and hemorrhage, and a reduction in stillbirths by 36 %. An overall decrease in maternal mortality ratio from 496 per 100,000 live births in 2007 to 328 per 100 000 in 2009 was also reported.

5. Community mobilization, peer-based interventions, or support programs

Community mobilization (CM) interventions seek to empower communities to modify power dynamics and form sustainable environments for better health [85]. CM may improve equitable access to health care services through individual capacity building skills, advocacy, and community actions [86–88]. Similarly, peer-based interventions or peer support are becoming popular as a strategy to improve access to and utilization of maternal health care especially for pregnant women living with HIV (WLH) [89, 90].

In rural Malawi, coupling facility quality improvements and community activities involving the sensitization and mobilization of the women using participatory approaches was shown to enhance the quality of maternal care provided [91]. A community-based safe motherhood intervention by skilled birth attendants also reportedly promoted the utilization of obstetric care in rural Tanzania [92], while family meetings in addition to labor and birth notification in Ethiopia were associated with an increase in the uptake of institutional-based deliveries [73]. In Kenya, the community health strategy implemented by the government used community health workers to deliver maternal and newborn care through household visits to screen pregnant mothers for danger signs and to provide support for birth planning and referrals. The initiative was reported as having significantly increased ANC attendance among pregnant women from 39 to 62 % as well as deliveries by skilled birth attendants from 31 to 57 %. The program was also associated with increased HIV testing during pregnancy from 73 to 90 % [93].

Community mobilization efforts to sensitize women to deliver in health facilities and encourage participation of men in the ANC attendance and HIV testing were conducted in rural Tanzania [92, 94]. Women were taught to make birth plans including places of delivery, savings to offset costs, and preparations for complications during pregnancy, labor, delivery, and the postnatal period. Findings indicate that the intervention improved both the utilization of health units for delivery and postnatal care within one month of delivery, without any significant negative effect on providers’ and women’s satisfaction with the ANC they provided or received respectively. Women in the intervention were shown to have discussed most elements of the birth plan with their providers, including how to recognize danger signs, identifying a delivery place, and making financial and transport arrangements for delivery. Women in the intervention arm were shown to be 16.8 % more likely to deliver in a health unit than women in the control arm and were 22.7 versus 2.2 % more likely to attend postnatal care within the first 48 h.

Other studies conducted in Burkina Faso, Uganda, and Zambia also found that community mobilization increased the level of health information about danger signs and risk factors in pregnancy and first ANC visits and health facility use and deliveries [54, 95, 96]. Community mobilization also reduced perinatal mortality rates. Similarly, peer mentor interventions by WLH on pregnant WLH at the antenatal and primary health care clinics were linked to significantly higher likelihood of completion of both maternal and infant ARV therapy regimens, increased adherence to all PMTCT tasks through 1.5 months after delivery, increased likelihood of asking partners to test for HIV, and lower likelihood of reporting depressed mood [90].

6. Home visits and counseling by health care workers

Home visits by health care workers, and usually community health workers (CHWs), is another strategy used to promote access to maternal health care in SSA for underserved communities [97–99]. In South Africa, home visits by CHWs to mothers in their neighborhood [89] reportedly improved adherence to condom use among pregnant WLH [100]. Similarly, volunteer peer counselor health education by women’s groups was shown to have decreased MMR by up to 52 % in poor rural populations in Malawi [101]. Birth plan counseling and health education during ANC were associated with increased uptake of skilled delivery and post-delivery care in Tanzania without negatively affecting women’s and providers’ satisfaction with available ANC services [94]. Job aids which are pictorial or illustrated cards used in counseling by nurses and midwives in Benin were said to be positively perceived by providers and pregnant women and associated with significant improvements in birth preparedness, danger sign recognition, safe and hygienic delivery, and even newborn care [102].

7. Emergency transportation, communication, and referrals

An intervention that provided free-of-charge 24-h ambulance and communication services between patients and health care providers reportedly increased access to and utilization of maternal health services, particularly caesarean delivery services from 0.57 to 1.21 % in Uganda. Also reported was a 50 % increase in the number of hospital deliveries, with a slight reduction in the average hospital stillbirths [103]. In South Africa, an inter-facility transportation scheme was associated with decreased institutional-based maternal mortality from 279 per 100,000 live births before implementation of the intervention in 2011 to 152 per 100,000 live births during implementation in 2012 [104]. CURGO (Centre d’UrgenceGyneco-Obstetric), an emergency obstetric care patient transfer system in Burundi, involving the relocation of patients requiring emergency obstetric care from public hospitals to a referral facility set up by the Médecins Sans Frontières was said to have averted 74 % of maternal deaths in Kabezi district [105]. A maternity referral system that included basic and comprehensive emergency obstetric care, transportation to obstetric health services and community cost-sharing schemes in Mali [106] was associated with an increase in the number of women receiving emergency obstetric care and caesarean sections performed for absolute maternal indications from 0.13 to 0.46 %. Institutional deliveries also increased from 19 % at baseline to 39.4 % at the completion of the intervention. Overall, the intervention was said to have reduced the risk of death by half in women treated for an obstetric emergency, with nearly half [47.5 %] of the reduction in deaths attributable to fewer deaths from hemorrhage.

8. Prevention of mother-to-child transmission of HIV

Prevention of mother-to-child transmission (PMTCT) of HIV programs often come as a set of interventions and are reported to be highly effective in the eradication of vertical transmission of HIV [107, 108]. While PMTCT coverage and utilization has remarkably increased over the past decade in SSA, it is still far from the recommended targets [108]. Community mobilization, as previously discussed, was employed in Uganda to encourage male partners’ acceptance of HIV testing through couple attendance at ANC. Findings showed a 16 % improvement in the number of women who attended ANC together with their partners, while 95 % of male partners who attended the ANC together with their spouses were tested for HIV [54, 109, 110]. Training of health care workers to offer PMTCT services and equipping health facilities with supplies in Mali and Senegal was shown to have produced significantly better quality obstetric care results [110, 111]. The number of pregnant women attending first ANC visit was said to have increased 11-fold in Northern Uganda, while the number of male partners counseled, tested, and given results for HIV together with their wives at the first ANC visit also rose significantly. In Cote d'Ivoire, a significant rise in the number of pregnant women delivering in the health facilities was reported following the intervention [112].

9. Task shifting interventions

Task shifting has been shown to be effective in improving maternal health care provision for patients in LMICs, most notably in the administration of ARV therapy [113, 114], non-communicable disease management [115–117], and mental health care [118]. Task shifting interventions involve equipping a cadre of staff in the health care system with the appropriate skills to provide services that would otherwise be provided by higher cadre providers, who are often scarce. In some countries in SSA, non-physician clinicians (NPCs) have been used to fill various roles that are conventionally handled by physicians [119–121]. In Benin, antenatal counseling by lay nurse aides was shown to have resulted in improvements in maternal knowledge among women in prenatal care compared to those counseled by nursing midwives. It also reportedly improved birth preparedness and recognition of danger signs in pregnancy [122]. In Ethiopia, NPCs were said to have performed 63 % of emergency caesarean section deliveries, reducing the number of maternal and fetal deaths experienced and reducing the length of hospital stay for patients in the country [123].

### Limitations of the study

It is important to highlight few limitations in our strategy. Limiting selection of the papers for inclusion to those published in English in selected databases raises the possibility that we could have excluded relevant research published in other languages or not indexed in the selected databases. Also, gray literature was not included in this review. Lastly, we are not able to assess the extent to which geographical, temporal, health systems and patient risk profiles affected maternal outcomes or confounded interpretations.

## Conclusions

Most of the non-drug interventions identified in this review targeted health systems capacity strengthening, including upgrading of the infrastructure with new construction and facelifts; provision of equipment and supplies to enhance maternal health care service provision; and training of health care workers to impact specialist skills for emergency obstetric care, criterion-based clinical audits and maternal death reviews, and PMTCT. Promotion of safe motherhood—as a right—is of crucial importance [124]. Effective communication and emergency transport services and prompt referrals for emergency obstetric care may reduce maternal mortality by addressing the three delays occasioned by (1) deciding when to seek appropriate medical help for an obstetric emergency, (2) reaching an appropriate obstetric facility, and (3) receiving adequate care when a facility is reached [125]. Community mobilization was linked to increased community awareness about the importance of seeking and utilizing skilled maternal care, while financial incentives through provision of redeemable vouchers, fee exemptions and community-based health insurance schemes were shown to facilitate access to and utilization of maternal health care by underserved populations.

Interventions that targeted strengthening health systems through processes like building capacity of health workers at the facility and community levels were shown to have led to the development of human resources with appropriate skills and increased coverage for maternal health services. Consequently, they were associated with improvements in the quality of care provided and reduction in obstetric complications and maternal deaths in such communities. Task shifting interventions offer a good alternative to provision of maternal health care, since they often employ competent non-physician qualified persons who earn lower remunerations and incur lower training costs compared to physicians. However, a higher level of supervision may be required for such personnel to deliver the expected impact services.

There are several non-drug interventions implemented in SSA aimed at improving the quality of maternal health services and care. Evidence on the effectiveness of the non-drug interventions on maternal outcomes as well as quality of maternal health varies. There is a preponderance of single facility and community-based studies whose effectiveness was difficult to assess. Implementation of elaborate and comprehensive interventions that strengthen different sectors of the existing health care systems, both in the community and at the health facilities, coupled with a supportive policy environment has the potential to not only improve the quality of maternal health care in SSA but save the lives of many women. Africa, as a region, fell short of achieving the MDGs 4 and 5 but has an opportunity to achieve more with the newly launched SDGs especially if decision making is guided by robust scientific evidence. Essentially, efforts to improve the quality of care and health outcomes for women in SSA must be guided by evidence about what works.

## Abbreviations

ALSO, advanced life support in obstetrics; AMDD, averting maternal death and disability; ANC, antenatal care; ARV, anti-retroviral therapy; CBCA, criterion-based clinical audits; CBHI, community-based health insurance; CFA, case fatality rate; CHW, community health worker; CM, community mobilization; DHIS, district health information system; eCDSS, electronic clinical decision support system; EmOC, emergency obstetric care; IMNCH, integrated maternal newborn and child health; LMICs, low- and middle-income countries; MaNHEP, maternal health in ethiopia partnership; MCH, maternal and child health; MDG, millennium development goals; MDR, maternal death reviews; MMR, maternal mortality rate/ratio; NPC, non-physician clinician; OBA, output-based approach; P4P, payment for performance; PBF, performance-based financing; PCR, polymerase chain reaction; PMTCT, prevention of mother-to-child transmission of HIV; PPH, postpartum hemorrhage; PROSPERO, prospective register of systematic reviews; QUARITE, quality of care, risk management and technology in obstetrics; RCT, randomized controlled trials; SSA, sub-Saharan Africa; SDGs, sustainable development goals; SMI, safe motherhood initiative; SMS, short messaging service; WLH, women living with HIV

## Additional file

Additional file 1: PRISMA 2009 Checklist for the systematic review. (DOC 63 kb)
